# The impact of MITF expression on tumor-infiltrating lymphocytes in melanoma: Insights into immune microenvironment dynamics

**DOI:** 10.17305/bb.2025.12125

**Published:** 2025-02-18

**Authors:** Damir Vučinić, Matea Lekić, Gordana Žauhar, Gordana Zamolo

**Affiliations:** 1 Tumor Clinic, Clinical Hospital Centre Rijeka, Rijeka, Croatia; 2 Department of Oncology and Radiotherapy, University of Rijeka, Faculty of Medicine, Rijeka, Croatia; 3 Special Hospital Radiochirurgia Zagreb, Sveta Nedelja, Croatia; 4 Department of Medical Physics and Biophysics, University of Rijeka, Faculty of Medicine, Rijeka, Croatia; 5 Department of Pathology, University of Rijeka, Faculty of Medicine, Rijeka, Croatia; 6 Department of Pathology, Clinical Hospital Centre Rijeka, Rijeka, Croatia

**Keywords:** Cutaneous melanoma, microphthalmia-associated transcription factor, MITF, tumor-infiltrating lymphocytes, TIL

## Abstract

Melanoma progression is influenced by complex interactions between tumor cells and the immune microenvironment. This study examined the relationship between microphthalmia-associated transcription factor (MITF) expression and the immune microenvironment in primary melanoma using a modified classification of tumor-infiltrating lymphocytes (TILs) based on conventional BRISK categories. Archival formalin-fixed, paraffin-embedded tissue samples from 81 primary melanoma patients were analyzed via tissue microarray immunohistochemistry to assess MITF protein levels. TIL patterns were categorized into six groups, refining the traditional BRISK classification to distinguish between continuous and discontinuous infiltration, as well as peripheral vs intratumoral distribution. The analysis revealed that melanomas classified under the BRISK B category exhibited the highest MITF expression, often exceeding 50%. In contrast, tumors in the NON-BRISK and ABSENT TIL groups showed significantly lower MITF expression (mean values: 32.73% ± 16.98% and 22.00% ± 10.54%, respectively), with statistically significant differences (Kruskal–Wallis test, *P* ═ 0.027; modified classification, *P* ═ 0.011). Additionally, the presence of CD20^+^ B lymphocytes correlated with increased MITF expression (*P* ═ 0.009). MITF gene amplification was detected in 29% of cases, though its association with protein expression showed only a trend (*P* ═ 0.058). These findings highlight the complex interplay between MITF expression and TIL distribution in melanoma, suggesting that refined TIL classification may offer deeper insights into tumor immunobiology and help predict responses to immunotherapy.

## Introduction

The inflammatory response plays a critical role in carcinogenesis and melanoma progression, with tumor-infiltrating lymphocytes (TILs) serving as independent prognostic markers. The distribution, density, and activation state of TILs can significantly influence clinical outcomes, highlighting their importance in the tumor microenvironment [1, 2]. At the same time, the microphthalmia-associated transcription factor (MITF) is a key regulator of melanoma cell phenotype [3]. High MITF expression (MITFhigh) is generally linked to a differentiated, proliferative state, while low expression (MITFlow) is associated with a dedifferentiated, invasive, and apoptosis-resistant phenotype [4]. Microenvironmental factors, such as hypoxia, nutrient availability, and cytokines further modulate MITF levels, impacting tumor behavior and response to targeted therapies and immune checkpoint inhibitors. Recent studies have shown that the localization of TILs—particularly continuous lymphocytic infiltration along the tumor base, as defined by BRISK categories—is associated with improved survival outcomes [5]. Conversely, reduced MITF expression has been linked to immune evasion, potentially contributing to resistance against therapies like anti-PD-1 and anti-PD-L1 [3–5]. By refining the BRISK classification to more accurately capture variations in TIL distribution, our study aims to elucidate the complex interplay between MITF expression and the immune microenvironment in melanoma. We hypothesize that high MITF expression correlates with specific patterns of TIL infiltration within the BRISK category, reflecting a more immunogenic tumor microenvironment. Ultimately, this study seeks to enhance our understanding of melanoma immunobiology and inform prognostic and therapeutic strategies.

## Materials and methods

### Subjects in the research

The research was conducted at the Department of Pathology and Pathological Anatomy, Faculty of Medicine, University of Rijeka. The tissue samples consisted of archival formalin-fixed, paraffin-embedded (FFPE) specimens obtained from the same department. These samples were collected from melanoma patients who underwent surgical excision between 2017 and 2021. A specialist dermatopathologist carefully selected paraffin blocks containing a sufficient amount of tumor tissue. In total, the study included 81 primary melanoma tissue samples (Table 1). The research protocol involved reviewing clinical data and histopathologic sections to classify the samples based on the following criteria: Clark and Breslow classification; AJCC; TNM category; melanoma histopathology categorized according to the dominant mode of growth and invasion; tumor inflammatory infiltrate assessed using the BRISK category. The study included 39 primary cutaneous melanomas with a Breslow thickness < 1.5 mm and 42 with a Breslow thickness > 1.5 mm. Based on the clinical stage according to the AJCC classification, we grouped the cases into the following categories: category 1—clinical stages 0, I, IIa; category 2—clinical stages IIb, IIc; category 3 consisted of clinical stage III, while category 4 included initially metastatic melanomas of clinical category IV.

**Table 1:** Clinical and pathological characteristics of the examined group of patients with primary melanoma

	**Group of primary melanomas**	**Percentage**
	***n* ═ 81**	
*Gender*		
Women	23	28%
Men	58	72%
*Age at the time of diagnosis*		
Median (range)	67,0 (21–90)	
*Anatomical localization of melanoma*		
Torso	42	52%
Limbs	23	28%
Head and neck	16	20%
*Grading of melanoma according to Breslow (thickness)*		
(0.1–1.5 mm)	39	48%
(>1.5 mm)	42	52%
*The histopathology of melanoma*		
Lentigo maligna	9	11%
Surface spreading	29	35%
Nodular	43	54%
*Sentinel lymph node*		
Positive	29	36%
Negative	52	64%
*Categories based on clinical stage*		
CATEGORY 1—0, I, IIa	38	47%
CATEGORY 2—IIb, IIc	14	17%
CATEGORY 3—III	26	32%
CATEGORY 4—IV	3	4%
*Conventional BRISK categories*		
BRISK	32	40%
NON BRISK	44	54%
ABSENT	5	6%

### Tissue microarray (TMA) immunohistochemical analysis

Immunohistochemical analyses of MM tissue samples were performed using the TMA method, a technique that consolidates tissue samples from multiple paraffin blocks into a single standardized paraffin block. This approach allows for the simultaneous analysis of multiple samples under identical experimental conditions, conserving both antibodies and probes used in immunofluorescence techniques. More importantly, it preserves biological material for further research and potential histopathological reassessment. While analyzing the entire tumor is not possible, studies have shown a high concordance between TMA results and those obtained from full tumor sections. A pathologist marks necrosis-free tumor tissue on an H&E-stained section, and three 1-mm diameter cylinders of tumor or control tissue are extracted from the corresponding paraffin block using the MTA Booster OI device. These samples are transferred into a recipient paraffin block with 0.5 mm spacing between them. Healthy liver tissue serves as an orientation guide. After TMA block preparation, it is incubated overnight at 45 ^∘^C to homogenize the paraffin and embedded tissue. Sections (4 µm thick) are mounted on silane-coated glass slides and dried at 37 ^∘^C for 12 h. Deparaffinization is performed with xylene substitute (Tissue Clear, Sakura, UK) three times for 10 min each, followed by rehydration in 100%, 96%, and 70% ethanol (5 min each) and a 10-min rinse in distilled water. Slides intended for detecting lymphocyte-specific antigens (PD-1, CD8, CD4, CD3, CD20, and Foxp3^+^) and MITF are immersed in Tris/EDTA buffer (pH 9) and heated in a laboratory pressure cooker (Pascal, DakoCytomation) to 125 ^∘^C. After reaching this temperature, incubation continues for 30 s, followed by a 20-min cooling period at room temperature and a distilled water rinse. The slides are then ready for immunohistochemical staining, which is performed using an automated immunostainer (Dako Autostainer Plus, DakoCytomation, Glostrup, Denmark). The antibodies used are listed in Table 2. The percentage of MITF-positive tumor cells (nuclear staining) was determined by manually counting at least 100 cells across representative high-power fields. To ensure accuracy, IHC results from the routine diagnostic work-up, including HMB-45 staining, were referenced. All melanoma cases had been confirmed with HMB-45 at the time of diagnosis, ensuring reliable identification of melanocytic cells. During evaluation, the pathologist compared MITF-stained sections with corresponding H&E slides and archived HMB-45-stained slides. This cross-referencing minimized misclassification by leveraging established melanocytic markers, ensuring precise tumor cell percentage determination.

**Table 2:** Characteristics of antibodies used for immunohistochemical analysis

**Antibody, catalog number cat. number**	**Manufacturer**	**Dilution of antibodies**	**Unmasking technique**	**Positive staining**
**Anti-CD3 – antibody RB A-HU T CELL, A045201**	Dako, Cytomation, Denmark	1:100	Tris/EDTA, pH 9 Heating 125 ^∘^C—35 s, cooling 20 min	Cell membrane, %
**Anti-CD8 – antibody MO A-HU T CELL,CD8, C8/114B, M710301**	Dako, Cytomation, Denmark	1:100	Tris/EDTA, pH 9 Heating 125 ^∘^C—35 s, cooling 20 min	Cell membrane, %
**Anti-CD4 – 104R-16**	Cell Marque, Germany	1:100	Tris/EDTA, pH 9 Heating 125 ^∘^C—35 s, cooling 20 min	Cell membrane, %
**Anti-Foxp3^+^ – antibody [236A/E7] 250 ug - ab20034**	Abcam, UK	1:100	Tris/EDTA, pH 9 Heating 125 ^∘^C—35 s, cooling 20 min	Cell membrane, %
**Anti-CD20 – antibody MONOCLONAL DAKO-B CELL, L26 - M075501**	Dako, Cytomation, Denmark	1:100	Tris/EDTA, pH 9 Heating 125 ^∘^C—35 s, cooling 20 min	Cell membrane, %
**Anti-MITF – antibody MO A-HU – M362129**	Dako, Cytomation, Denmark	1:100	Tris/EDTA, pH 9 Heating 125 ^∘^C—35 s, cooling 20 min	Cell nucleus, %

### Evaluation of immunohistochemical preparations to determine the lymphocytes infiltrating the tumor

**Figure 1.**
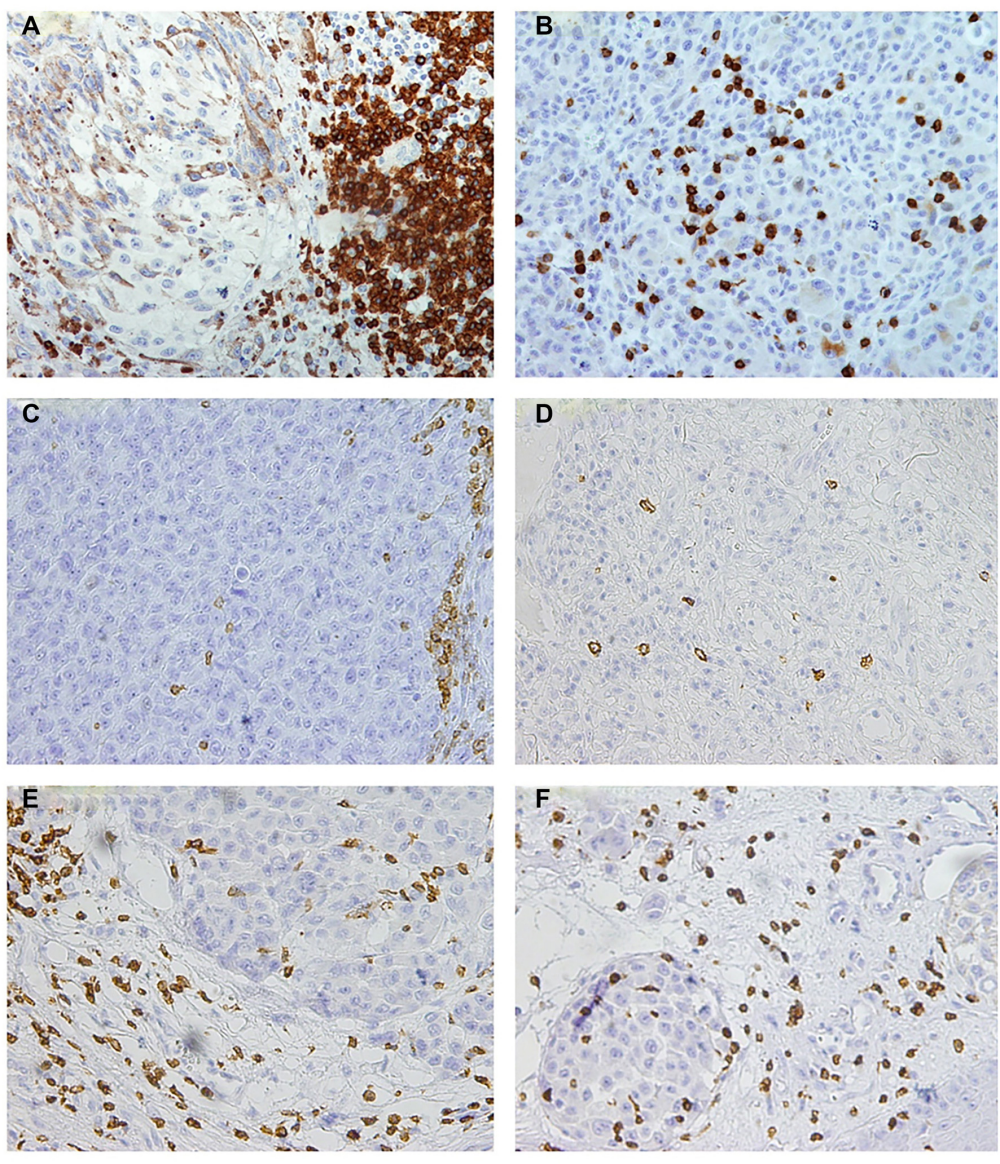
**Classification of TILs in melanoma by modified BRISK categories.** This figure illustrates the classification of TILs in melanoma based on BRISK categories. The categories include: (A) BRISK A—continuous lymphocytic infiltration at the tumor base with at least four rows of lymphocytes; (B) BRISK B—lymphocytic infiltration within the tumor tissue; (C) NON-BRISK A—discontinuous lymphocytic infiltration with fewer than four rows; (D) NON-BRISK B—focal lymphocyte distribution within the tumor; (E) ABSENT TIL A—infiltration distant from tumor tissue; and (F) ABSENT TIL B—localized TIL around blood vessels or areas of fibrosis. TIL: Tumor-infiltrating lymphocyte.

The control tissue for MITF expression analysis was obtained from a benign melanocytic nevus, while spleen tissue was used as the control for CD3, CD4, and CD8 immunohistochemistry, given its naturally high concentration of T lymphocytes expressing CD3 and CD8. For IHC analysis, the selected areas had to include both tumor tissue and surrounding inflammatory infiltration. TMA block preparation was followed by IHC staining for CD3, a marker used to identify T lymphocytes within the inflammatory infiltrate. Due to the heterogeneous patterns of lymphocytic infiltration, we implemented a modified classification system that refines the conventional BRISK categories (Figure 1). This modification aimed to more precisely distinguish variations in lymphocytic distribution, which may have biological and clinical relevance to melanocytic differentiation and immune response. Additional sections were analyzed to identify T lymphocyte subtypes and assess potential B lymphocyte infiltration. The number of stained cells was expressed as a percentage relative to the total T lymphocytes detected by CD3 IHC staining. Based on the presence of CD20^+^ lymphocytes, melanomas were classified into two categories, one of which included cases with B lymphocyte infiltration around the tumor tissue.

### DNA isolation and analysis of MITF amplification

For MITF gene amplification analysis, DNA was extracted from archival primary melanoma specimens embedded in paraffin blocks. Several microtome sections (5–10 µm thick) were mounted on glass slides, with one section stained with H&E for histological evaluation [6, 7]. A pathologist identified and marked tumor-rich areas devoid of necrosis and immune cell infiltration. These marked regions were microdissected from unstained sections using a sterile scalpel. DNA extraction was performed using the NucleoSpin Tissue kit (Macherey-Nagel, Düren, Germany) according to the manufacturer’s protocol. The quality and concentration of the extracted DNA were assessed using a Qubit 3.0 fluorometer with the Qubit DNA HS assay (Invitrogen, Carlsbad, CA, USA), yielding concentrations of 0.5–12 ng/µL. MITF gene amplification was analyzed via the SYBR Green real-time PCR method, following protocols by Garraway et al. (2005) [8] and Ugurel et al. (2007) [9]. Each sample was amplified in duplicate using SYBR Premix Ex Taq (Perfect Real Time) from Takara Bio Inc. (Shiga, Japan). MITF-specific primers were used, with LINE1—a repetitive element with a stable copy number in normal and cancer cells—serving as the reference gene. Relative MITF and LINE1 copy numbers were normalized against genomic DNA from leukocytes of 10 healthy donors, which served as a diploid calibrator. The primer sequences for MITF were as follows: forward 5′-GACCCACCTCGAAAACCCCACCAAGT and reverse 3′-GTGGGAATCATATTCAACAGACAAG, producing a 293 bp product, while the LINE1 primers consisted of forward 5′-AAAGCCGCTCAACTACATGG and reverse 3′-TGCTTTGAATGCGTCCCAGAG, yielding a 150 bp product.

The amplification rate (AR) for tumor DNA (AR Tu-DNA) was calculated using the formula:

**AR Tu − DNA ═ 2 − (−ΔΔCT),**

where ΔΔCT (MITF) ═ [CT (MITF; tumor) − CT (LINE1; tumor)]/[CT (MITF; calibrator) − CT (LINE1; calibrator)].

To account for the proportion of tumor cells, the percentage of cells stained for MITF was used to adjust the AR in tissue (AR Tu-T), calculated as:

**AR Tu − T ═ (AR Tu-DNA − 1 + PTS)/PTS**.

An increase of 0.5 in AR indicates the gain of one MITF copy. All PCR reactions were duplicated on an ABI 7500 Real-Time PCR System (Applied Biosystems, USA), and the amplification cut-off was set at four copies, corresponding to an AR of 2.

### Statistical analysis

The database was created in MS Excel, while data processing and analysis were conducted using the statistical software Dell Statistica v.12 (Dell Inc., 2015, software.dell.com). Data were entered into a spreadsheet, and appropriate statistical tests were selected based on measurement scale, dependency, number of groups, data distribution, and sample size. Parametric or non-parametric tests were applied accordingly, with statistical significance set at *P* < 0.05. To summarize the data, we used classical descriptive statistics. Due to the non-normal distribution of certain datasets, we employed non-parametric tests, including the Kruskal–Wallis ANOVA, which allows for the comparison of multiple independent groups without assuming normality. This approach provided a more nuanced interpretation of MITF expression across BRISK categories. Additionally, descriptive statistics and Mann–Whitney tests were used to further explore relationships between clinical features and immunohistochemical findings.

### Ethical statement

The study protocol was approved by the Institutional Review Board of Clinical Hospital Centre Rijeka (approval number: 003-05/21-1/67) and all participants provided written informed consent by the Declaration of Helsinki.

## Results

### Analysis of MITF protein expression and the examined clinical-pathological features of melanoma

**Figure 2.**
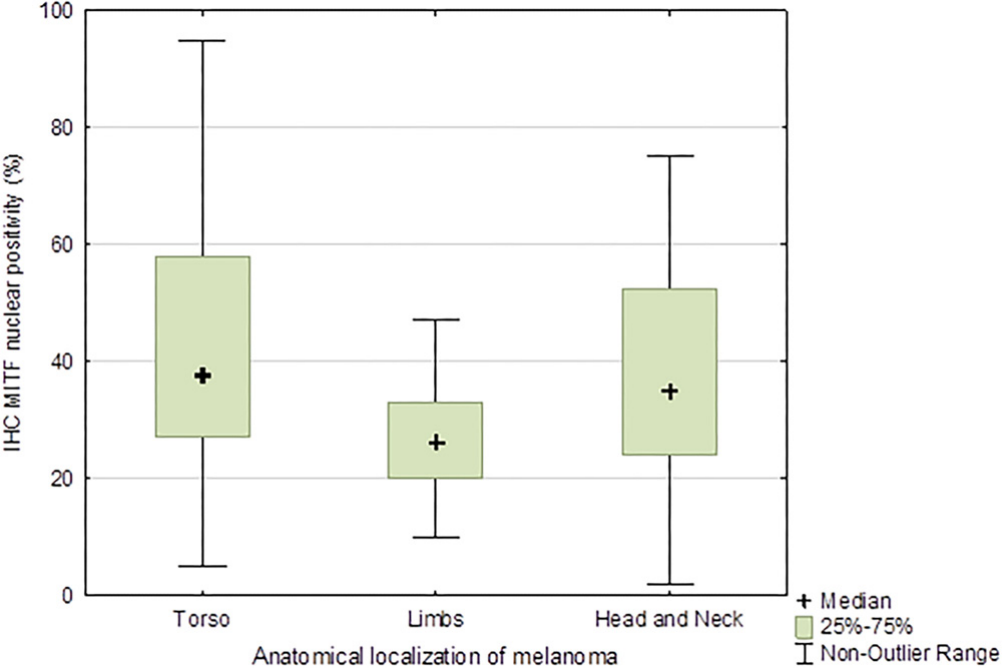
**Box-and-whisker plot of MITF expression by anatomical location of melanoma.** This box-and-whisker plot highlights statistically significant differences in MITF protein expression across melanomas in different anatomical regions (*P* ═ 0.03). The groups include melanomas on the torso, extremities, and head/neck, with marked medians and interquartile ranges. MITF: Microphthalmia-associated transcription factor.

**Figure 3.**
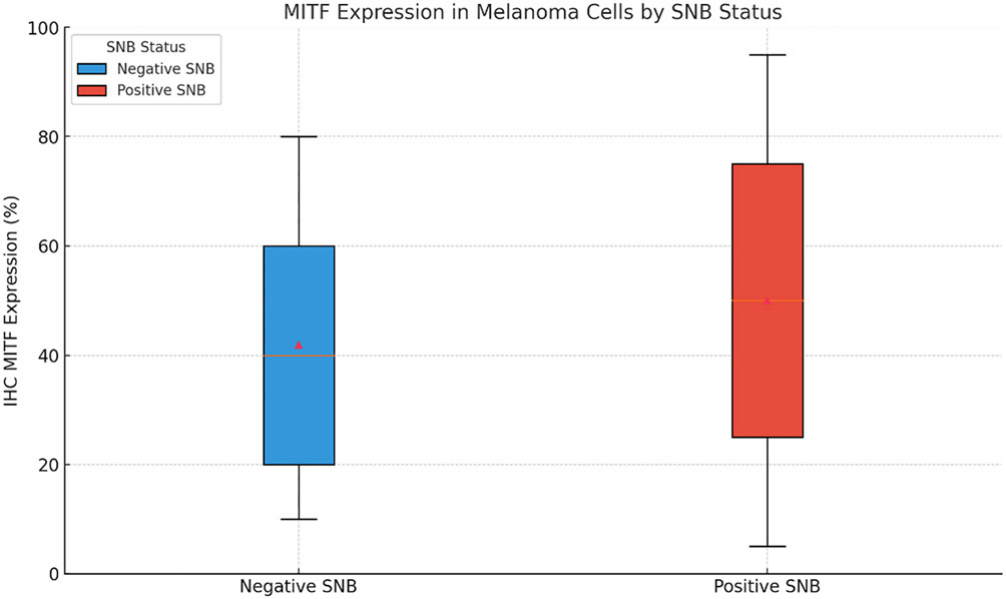
**Box-and-whisker plot illustrating significant differences in MITF protein expression in melanoma tumor cells by SNB status.** Positive SNB cases show higher variability in expression. The median and interquartile ranges are highlighted. *P* ═ 0.022. MITF: Microphthalmia-associated transcription factor.

We analyzed MITF protein expression across various clinical and pathological subgroups of melanoma. The median MITF expression was 33% (range: 2%–95%). Expression varied significantly by the anatomical location of the primary melanoma (Figure 2), with distinct patterns observed in melanomas of the torso and limbs. Additionally, MITF expression was significantly higher in cases where melanoma had spread to the sentinel lymph node (*H* ═ 5.21, *P* ═ 0.022) (Figure 3). When stratified by Breslow thickness, melanomas <1.5 mm had a median MITF expression of 24% (95% CI: 2%–68%), while those >1.5 mm had a median of 33% (95% CI: 12%–92%), a statistically significant difference (*P* ═ 0.032). MITF levels were also significantly higher in advanced clinical stages, with melanomas in stages 0–IIa showing a median expression of 14% (95% CI: 2%–52%) compared to 47% (95% CI: 14%–87%) in stages III–IV (*H* ═ 8.41, *P* ═ 0.03). No other statistically significant differences were observed.

### The presence of CD8^+^, CD4^+^, and Foxp3^+^ T lymphocytes in TIL and researched clinical and pathological features of melanoma

**Figure 4.**
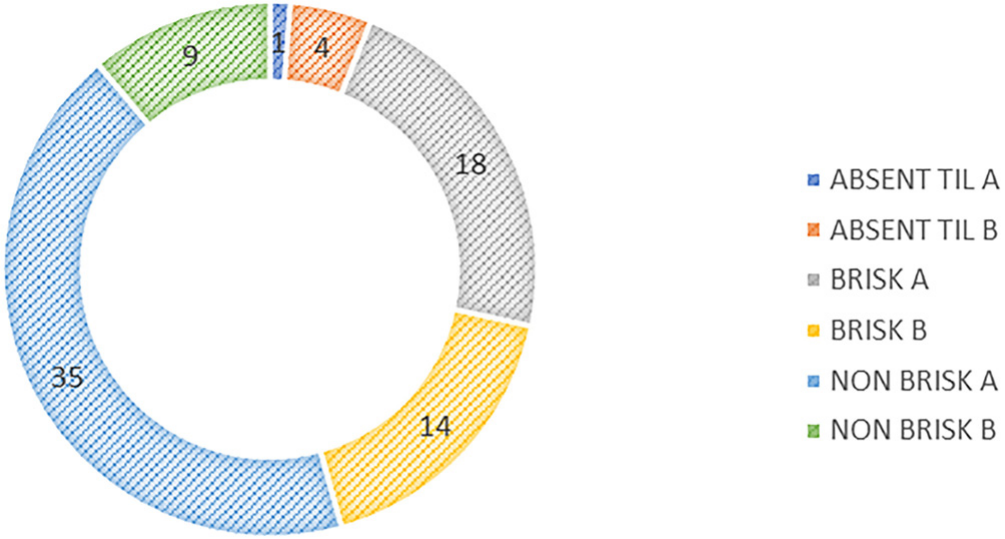
**Distribution of melanomas by specific modified BRISK CATEGORIES.** This histogram illustrates the distribution of melanomas across BRISK categories. The NON-BRISK A Category comprises 43% of all samples, with other categories representing smaller proportions. TIL: Tumor-infiltrating lymphocyte.

After IHC staining of the CD3 molecule, we categorized TILs in the collected samples and confirmed that 43% of all melanoma inflammatory infiltrates belonged to the NON-BRISK A category (Figure 4). Based on IHC identification of T lymphocytes via CD3 staining, we analyzed the proportions of CD8^+^ and CD4^+^ lymphocytes in TILs across all primary melanomas in this study. The median expression of CD8^+^ lymphocytes in TILs was 60% (interquartile range: 1%–90%), while CD4^+^ lymphocytes had a median expression of 20% (interquartile range: 1%–90%). Additionally, we examined Treg lymphocytes and found that their Foxp3^+^ marker had a median positivity of 1%, ranging from 0% to 70%. No statistically significant correlation was observed between CD8^+^/CD4^+^ lymphocytes and MITF expression (*H* ═ 7.73, *P* ═ 0.172; *H* ═ 6.94, *P* ═ 0.225). While these results aligned with previous studies (e.g., Mishra et al., 2019), they highlighted the complexity of immune–melanoma interactions, suggesting that other immune or tumor-related factors may play key roles in modulating MITF expression, warranting further mechanistic investigation. We also found no dominant presence of Treg lymphocytes in any TIL category (*H* ═ 3.01, *P* ═ 0.697). However, CD4^+^ lymphocyte expression was significantly higher in lentigo maligna melanomas compared to other subtypes (*H* ═ 6.76, *P* ═ 0.034), with a mean value of 46% (95% CI: [1%, 59%]). No statistically significant differences were observed in the expression of the tested lymphocytes across other clinical feature groups.

### Analysis of MITF protein expression about the BRISK category of tumor inflammatory infiltrate

**Figure 5.**
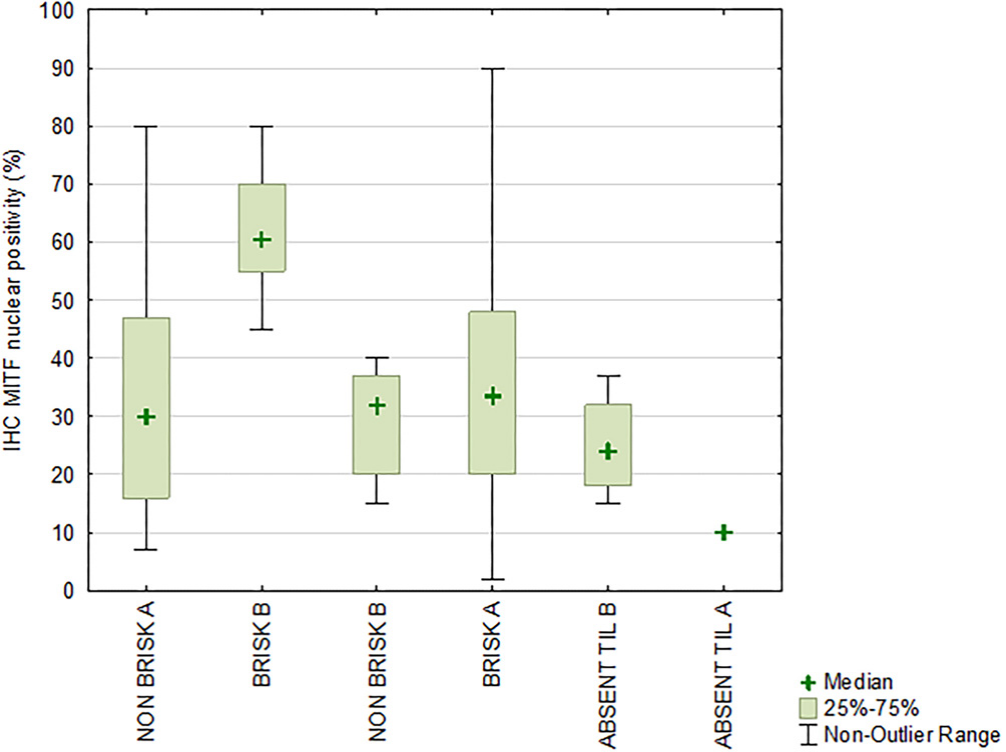
**Comparison of MITF protein expression across modified BRISK categories.** This box-and-whisker plot compares MITF protein expression in different BRISK categories of tumor-infiltrating lymphocytes (*P* ═ 0.011). The expression is notably higher in the BRISK B category (> 50%) compared to other categories. The figure also indicates significantly lower MITF expression when lymphocytes are located at the tumor edges. MITF: Microphthalmia-associated transcription factor; TIL: Tumor-infiltrating lymphocyte.

Our analysis reveals a statistically significant association between conventional BRISK categories and MITF protein expression in tumor cells (Kruskal–Wallis ANOVA test, *P* ═ 0.027). Notably, tumors classified as BRISK exhibit the highest mean MITF expression (44.16 ± 24.10%), with values ranging from 2% to 95%. In contrast, NON-BRISK tumors show a lower mean expression (32.73 ± 16.98%), while ABSENT TIL cases display the lowest MITF expression (22.00 ± 10.54%). Furthermore, the comparison of IHC MITF expression across modified BRISK categories remains statistically significant (Kruskal–Wallis ANOVA test, *P* ═ 0.011). Notably, MITF protein expression in tumor cells is almost always greater than 50% when TIL falls within the BRISK B category (Figure 5). Conversely, when lymphocytes are positioned along the edges of the melanoma, MITF protein expression is significantly lower.

### Analysis of MITF protein expression about the presence of CD20^+^ B lymphocytes in TIL

**Figure 6.**
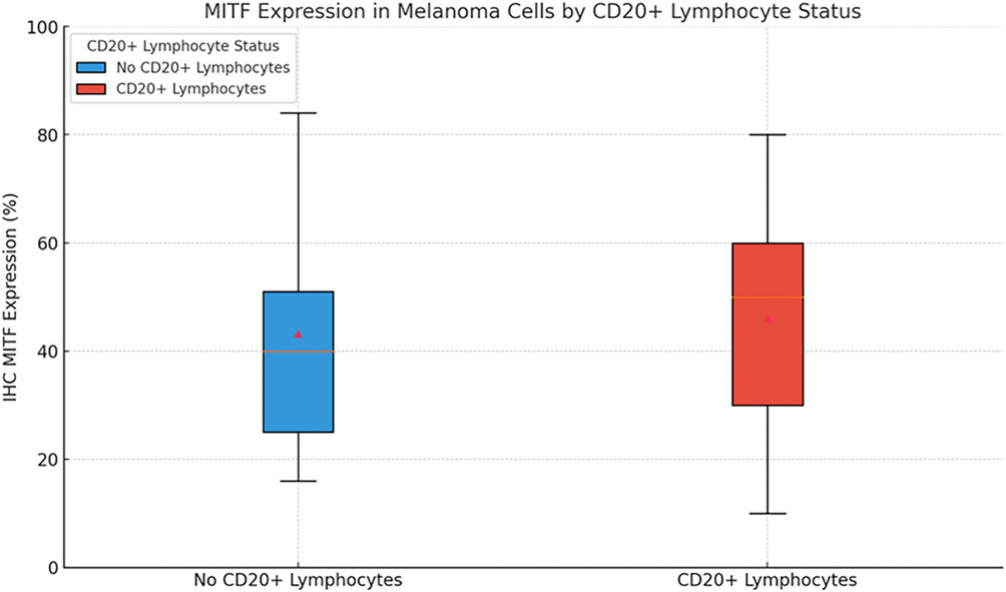
**Box-and-whisker plot showing significant differences in MITF protein expression in melanoma tumor cells by CD20^+^ lymphocyte status.** Cases with CD20^+^ lymphocytes had significantly higher expression (median 51%, 95% CI: [16%, 84%]) compared to cases without (*P* ═ 0.009). MITF: Microphthalmia-associated transcription factor.

We performed IHC staining for the CD20 molecule on all primary melanoma samples. Given the expected low proportion of B lymphocytes among TILs, we classified the biopsies into two groups based on their presence. CD20^+^ B lymphocytes were identified in the TILs of 18 (22%) examined melanomas. Notably, in eight of these positive cases, the TIL category was classified as BRISK A. MITF expression in cases with CD20^+^ lymphocytes was 51% (95% CI: [16%, 84%]), significantly higher than in cases without CD20^+^ cells (*P* ═ 0.009) (Figure 6).

### Molecular analysis of MITF gene amplification in primary melanoma samples

We successfully isolated a sufficient amount of DNA from all samples for analysis. Amplification was confirmed in 24 samples (29%). Notably, statistical significance was not achieved for the IHC value of MITF protein expression, though a trend was observed (*P* ═ 0.058). Other analyzed parameters, including clinical and pathological features and TIL categorization, showed no statistically significant differences based on the presence or absence of MITF amplification.

## Discussion

The results of this study provide valuable insights into the role of MITF expression in melanoma and its relationship with TILs. While our findings align with prior research, they also highlight novel aspects that contribute to the current understanding of melanoma’s immune microenvironment. In our research, we demonstrated an association between MITF protein expression and a specific category of TILs. Notably, almost all melanomas with TILs classified as BRISK B exhibit MITF expression levels above 50% [10, 11]. This aligns with findings from Balatoni et al., who reported that increased TIL levels localized at the tumor periphery were associated with improved survival in melanoma patients [1, 5]. Additionally, de Moll et al. [12] studied a cohort of 94 patients and found that TILs were strongly correlated with survival, but only in cases of ulcerated melanoma. While our study did not specifically analyze ulcerated melanoma subsets, the variations in TIL categories and their correlation with MITF expression suggest a complex interplay that may warrant further stratification based on tumor subtype and clinical features. Heterogeneity among patient populations and selection criteria complicates the evaluation and comparison of different conclusions. For instance, studies have examined melanoma patients across all stages and from different countries, confirming that the BRISK category of TILs is associated with better overall survival [13]. However, Mishra et al. [14] reported no significant association between BRISK-classified TIL groups and overall survival. While the BRISK classification is widely used, modifications introduced in this study have altered key categorization criteria, complicating direct comparisons with existing literature. These modifications aimed to improve specificity in categorizing TILs, but they may also impact reproducibility by introducing inconsistencies when comparing findings across studies. Previous studies have explored BRISK-classified TILs in relation to checkpoint inhibitor therapy response [15]. In our study, we did not confirm a direct connection between CD8^+^ lymphocytes and MITF protein expression. However, the presence of cytotoxic lymphocytes within melanoma tumors likely plays a crucial role in regulating MITF expression [3, 4, 15]. The observed lack of a statistically significant relationship between CD8^+^ lymphocytes and MITF expression underscores the complexity of immune–tumor interactions. While CD8^+^ T cells are well known for their cytotoxic role in anti-tumor immunity, our findings suggest that additional factors within the tumor microenvironment influence MITF expression. This aligns with emerging evidence that immune responses in melanoma are shaped by a combination of cellular and molecular dynamics, including the spatial distribution of immune cells, cytokine signaling, and the phenotypic plasticity of melanoma cells. For instance, Mishra et al. [14] demonstrated that CD8^+^ T cell density varies depending on tumor subtype and immune evasion mechanisms, which may explain why our analysis did not find a direct link. Additionally, the dedifferentiated phenotype associated with MITF-low tumors may inherently reduce CD8^+^ lymphocyte recruitment or activation, further complicating this relationship. CD20, a B cell marker encoded by the MS4A1 gene, is a phosphoprotein embedded in the lymphocyte membrane. It is expressed in B lymphocytes and typically disappears when B cells mature into plasma cells. The engagement of tumor-reactive B lymphocytes may be crucial for generating strong, long-term T cell responses against cancer. In cervical cancer, CD20^+^ TILs have been associated with a lower recurrence rate. Furthermore, Willsmore et al. emphasize that B lymphocytes are not merely “observers” in melanoma immunotherapy but may play an active role in shaping anti-tumor responses. The role of T lymphocytes in immune activation appears to be a key factor in achieving a strong response to immunotherapy. Our analysis revealed that CD20^+^ B lymphocytes are associated with higher MITF expression levels. This observation aligns with findings by Willsmore et al., who highlighted the active role of B lymphocytes in enhancing immune responses and their potential interplay with MITF-driven mechanisms [16–19]. Regulatory T (Treg) lymphocytes play a crucial role in maintaining immune homeostasis. Several studies have examined correlations between melanoma prognostic factors—such as Breslow thickness and mitotic index—and the expression of FOXP3+ regulatory T cells and dendritic cells within the tumor stroma. These studies suggest that FOXP3+ could serve as a valuable therapeutic target in melanoma, as inhibiting regulatory T cells may prevent tumor cells from evading the host’s immune defenses [20]. Moreover, research has shown that in melanomas with a vertical growth phase, the density of CD8^+^ lymphocytes adjacent to the normal dermis significantly impacts patient outcomes. Mortality rates varied from 81% in cases with low CD8^+^ density to 20% in cases with high CD8^+^ density [21]. These findings support our conclusion that both the location and density of TILs play an essential role in regulating potential biomarkers for melanoma therapy. MITF protein expression is crucial for melanoma cell survival and proliferation [4, 6, 9]. The BRAF V600E oncogene influences MITF activity in two opposing ways: it enhances MITF degradation through ERK-mediated phosphorylation, reducing its protein levels, while simultaneously promoting MITF transcription via the activation of BRN-2. Despite some degradation in the proteasome, this dual mechanism ensures sufficient MITF remains to support cell proliferation [22]. MITF has also been explored as a potential biomarker in immunotherapy. Low MITF expression has been associated with resistance to immune checkpoint inhibitors, particularly in melanoma treated with BRAF/MEK inhibitors. This finding supports the idea that MITF-low tumors may adopt a dedifferentiated phenotype, potentially evading immune recognition. The integration of MITF expression analysis into therapeutic decision-making could help stratify patients who are more likely to benefit from immunotherapy. However, challenges remain in standardizing MITF measurement and establishing its prognostic value across diverse clinical settings. Our results suggest that incorporating MITF expression analysis into treatment planning could improve predictions of immunotherapy outcomes, particularly in cases where TIL characteristics alone are less informative [8, 22]. Although genetic alterations, such as MITF mutations and amplifications have been identified in melanoma, MITF activity is more likely influenced by microenvironmental signaling, key epigenetic states, and upstream pathway modifications [4]. These factors collectively shape MITF transcriptional activity, contributing to distinct cellular behaviors. Several limitations of our study should be acknowledged. The relatively small sample size may affect the generalizability of our findings, particularly in subgroup analyses involving BRISK categories and lymphocyte subsets. Additionally, the lack of longitudinal data prevents us from drawing definitive conclusions about MITF expression as a prognostic biomarker. Future research should incorporate longitudinal studies to assess dynamic changes in MITF expression throughout disease progression and treatment. Further investigation into the molecular mechanisms linking MITF expression to immunotherapy resistance—especially in conjunction with genetic and epigenetic profiling—will be essential for refining its clinical utility.

## Conclusion

This study reinforces the significance of MITF expression in the melanoma immune microenvironment and its potential as a biomarker for therapeutic response. By contextualizing our findings within the existing literature, we underscore the need for further investigation into MITF and related pathways to enhance melanoma management and improve patient outcomes.

## Data Availability

All data generated or analyzed during this study are included in this published article.
